# Elevating Testosterone and Androstenedione Produces Temporary Sex‐Dependent Variation in Japanese Quail (*Coturnix japonica*) Embryonic Development

**DOI:** 10.1002/jez.70024

**Published:** 2025-08-08

**Authors:** S. K. Winnicki, M. E. Hauber, R. T. Paitz, W. M. Schelsky, T. J. Benson

**Affiliations:** ^1^ Department of Evolution, Ecology, and Behavior, School of Integrative Biology University of Illinois at Urbana‐Champaign Urbana Illinois USA; ^2^ Program in Ecology, Evolution, and Conservation University of Illinois at Urbana‐Champaign Urbana Illinois USA; ^3^ Illinois Natural History Survey, Prairie Research Institute University of Illinois at Urbana‐Champaign Champaign Illinois USA; ^4^ School of Environment and Natural Resources, College of Food, Agricultural, and Environmental Sciences The Ohio State University Columbus Ohio USA; ^5^ Advanced Science Research Center and Programs in Biology and in Psychology Graduate Center of the City University of New York New York New York USA; ^6^ School of Biological Sciences Illinois State University Normal Illinois USA; ^7^ The Grainger College of Engineering University of Illinois at Urbana‐Champaign Urbana Illinois USA

**Keywords:** androstenedione, embryo, growth, hormones, steroid, testosterone, yolk

## Abstract

Vertebrate growth rates may respond to maternally supplied steroids, yet injections of individual hormones into avian eggs of different species have produced inconsistent results. This might be because diverse steroids' concentrations are naturally correlated in yolks, injections are not elevating hormones at critical times in development, or injected hormones are rapidly metabolized by the developing embryo. To explore these alternatives, we injected naturally occurring levels of androstenedione (A4) and testosterone (T) or an oil control into fertile Japanese Quail (*Coturnix japonica*) eggs at the onset (Day 0) and/or on Day 6 of incubation. We measured the size of the embryos at two points in development (Days 6 and 15), testing for an effect of the hormone injections and the embryos' sex determined by molecular markers. Additionally, we quantified the concentrations of 27 yolk steroid hormones at five points in development (Days 0, 1, 6, 7, and 15). Ultimately, the A4 + T injection did not elevate yolk concentrations of these hormones 24 h after treatment, nor did it influence concentrations of other steroids in the yolk. Still, initial (Day 0) A4 + T injections reduced the eye diameter and leg length of female embryos and reduced the mass of male embryos on Day 6. The double hormone treatment (A4 + T on Days 0 and 6) produced embryos with larger beaks and wings but reduced mass measurements on Day 15 relative to the oil‐oil control, regardless of sex. This suggests that embryonic growth responds to elevated levels of A4 and T at the beginning of incubation and in a sex‐ and structure‐specific manner.

## Summary

- Experimentally elevating two androgen steroids before the onset of avian egg incubation altered embryonic size in a sex‐ and structure‐specific manner.
- Injections of testosterone and androstenedione did not produce detectable elevations in yolk androgen concentrations 24 h after injections, suggesting rapid metabolism.
- Embryos exposed to injections before and 6 days after the onset of incubation had longer beaks and wings but a lighter mass than embryos exposed to oil control injections.

## 1 Introduction

Juvenile organisms vary in their growth rates and developmental trajectories (Dmitriew 2011); this variation exists not only among species (Poorter 1989; Chown and Gaston 2010) but also within species (Gebhardt‐Henrich and Richner 1998; Segura and Palacio 2021). The consequences of variation in growth rate can be immense, with slower‐growing organisms being more prone to predation risk (Remeŝ and Martin 2002) and suffering more from competition for resources (Arendt and Wilson 1997), while rapidly growing organisms may face developmental abnormalities (Reimer et al. 2017) and have shorter lifespans (Gabriela Jimenez 2018; Monaghan and Ozanne 2018). Understanding the mechanisms that give rise to variation in growth rate can help explain the diversity we observe in life history strategies in both wild populations and domestic animals.

Organismal development is often controlled by hormonal inputs (Leet et al. 2011). Most studies on how steroid hormones control development have focused on the role of the embryonic gonads as a steroid input that regulates the differentiation of peripheral tissues in a sex‐specific manner depending on whether testes or ovaries are present (Adkins 1979). A fundamental principle underlying this field of study is that there are critical periods during development when tissues are sensitive to the effects of steroids such that exposure before or after this period fail to influence tissue development (Adkins 1979; Nakabayashi et al. 1998). However, maternally derived steroids have been identified as a distinct hormonal input that can influence offspring development in a variety of ways. In placental mammals, for example, these hormones can come from the offspring's maternal parent, through the placenta before birth (Calvo et al. 1992; Costa 2016) and post‐natal mammary secretions (Mazzocchi et al. 2019). Developing oviparous organisms, however, are typically limited to the deposited resources already inside the fully formed egg. Since the discovery of adaptive variation in yolk hormones of avian eggs (Schwabl 1993), maternally deposited yolk hormones have proven to be a tantalizing source of developmental variation in birds (Gil 2008; Groothuis and Von Engelhardt 2009), with research linking hormones to the development of the offspring inside the egg (Darras 2019), to the post‐hatch development and behavior of the offspring (Adkins‐Regan et al. 2013), and even to long‐term effects on mature organisms (Ruuskanen and Laaksonen 2010). Despite the progress made in understanding the roles of yolk hormones in avian development, the full impact of the suite of hormones within an egg on the growth of young birds remains unknown (Groothuis et al. 2019).

Testosterone is a frequently studied androgen steroid hormone present in avian egg yolk (Schwabl 1993; Groothuis et al. 2005; Okuliarová et al. 2009). The accumulation of testosterone in yolk may be affected by the environmental conditions experienced by the laying female (e.g., light conditions [Schwabl 1996a]), the availability of high‐quality mates (Gil et al. 2004), and competitive environments (Bentz, Becker, et al. 2016), and could allow parents to prime their offspring for prevailing conditions (e.g., brood parasitism (Hahn et al. 2017), drought (Pilz et al. 2004), sibling competition (Eising et al. 2001)). Among species, higher testosterone content has been linked to faster embryonic development in passerine birds (Schwabl et al. 2007), but direct testosterone injections have produced equivocal results on within‐species variation in embryonic growth. For example, testosterone injections increase embryonic growth in House Finches (*Carpodacus mexicanus*, Navara et al. 2006) but suppressed the growth of female chicken (*Gallus gallus domesticus*) embryos with no measurable impact on male embryos (Henry and Burke 1999) and produced no effect on the growth of Blue‐breasted Quail (*Excalfactoria chinensis*) embryos (Andersson et al. 2004). Despite the interest in investigating the effects of testosterone on growth, the results of testosterone‐injection experiments have not illustrated that testosterone has a predictable effect on developing birds.

Testosterone is not the only androgen present in avian egg yolks; in many species androstenedione (A4) is present in higher concentrations in unincubated eggs (Gil and Faure 2007), yet its effects are less commonly studied than the effects of testosterone (Groothuis et al. 2005). Higher naturally occurring A4 concentrations have been linked to larger sizes at hatch and greater recruitment to natal locations in Collared Flycatchers (*Ficedula albicollis*) (Hegyi et al. 2011), but passerine species with higher A4 concentrations do not develop faster than species with lower A4 concentrations (Schwabl et al. 2007). Androstenedione injections reduced hatchling Spotless Starling (*Sturnus unicolor*) body mass relative to tarsus size (Muriel et al. 2013), led to increased skeletal growth in female chicken hatchlings but not males (Benowitz‐Fredericks and Hodge 2013), and did not impact Japanese Quail (*Coturnix japonica*) hatchling growth (Hegyi and Schwabl 2010). Like testosterone, the effects of elevated androstenedione on growth are equivocal.

Androstenedione and testosterone do not exist in eggs in isolation; concentrations of these two androgens are frequently positively correlated (Hegyi et al. 2011). Furthermore, A4 can be metabolized into testosterone during development via 17β‐hydroxysteroid dehydrogenase (17β‐HSD), which is already present in chicken embryos by the second day of incubation (Bruggeman et al. 2002). Therefore, some past research has also investigated the impact of simultaneously elevating A4 and T with injections into eggs (Muriel et al. 2015). While testosterone or A4 injections alone into eggs increased the skeletal growth of Spotless Starlings post‐hatch, the combined A4 + T treatment suppressed growth, potentially indicating antagonistic interactions between these androgens (Muriel et al. 2013). Combined A4 + T treatments increase embryonic development periods in some species (American Kestrels: *Falco sparverius*, Sockman and Schwabl 2000), but decrease it in others (Black‐headed Gulls: *Chroicocephalus ridibundus* (Eising and Groothuis 2003) and European Starlings *Sturnus vulgaris* (Müller and Eens 2009)). While previous research combining A4 and T have again produced complex results, studying the androgens in combination may better reflect natural variation in these correlated steroid hormones.

The possible effects of hormones on developing embryos are further complicated by the rapid metabolism and conversion of these hormones within the egg (Paitz and Bowden 2013). Within 12 h of the onset of incubation in European starlings, injected testosterone is already being converted to etiocholanolone, while etiocholanolone injections had no impact on early embryonic development, suggesting that testosterone conversion and/or metabolism may render it inert early in the development of the offspring (Campbell et al. 2020). However, T and A4 can also be converted into other hormones, such as estrogens (Bruggeman et al. 2002; Kamata et al. 2004), which could have downstream effects on development (Addison and Rissman 2012; Bentz, Sirman, et al. 2016). The potential rapid conversion of hormones at the onset of incubation creates a situation where it is important to understand how the rate of conversion and/or metabolism relates to the development of tissue sensitivity. Work on sexual differentiation of reproductive behavior in Japanese Quail established that embryos are sensitive to testosterone injected between days 10 and 12 (Adkins 1979), but very little work has been done on embryonic sensitivity to androgens before incubation day 10 when most exposure to maternally deposited androgens is likely taking place. The timing of hormone injections and the embryo and embryonic membranes' potential to metabolize these hormones may further impact our understanding of the effects of yolk steroids on avian development.

Here we experimentally tested the effect of combined T and A4 injections on embryonic growth and yolk hormone milieu at two developmental benchmarks (pre‐incubation Day 0 and Day 6 of incubation) by injecting a hormone cocktail with biologically‐relevant levels of A4 + T or an oil control into fertile quail eggs. Initial pre‐incubation (Day 0) injections mimic higher levels of maternally derived androgens; since early exposure to steroids has been shown to impact embryonic development (Elbrecht and Smith 1992), we anticipated that Day 0 injections could have impacts on embryo growth and yolk hormones throughout development. Previous research showing an impact of injected androgens on quail development (e.g., Adkins 1979) did not inject hormones to mimic maternally derived hormone exposure but rather injected eggs later in development, when embryos would be exposed to endogenously produced steroids. As maternally derived hormones may be rapidly metabolized or converted, possibly as a mechanism for offspring to resist maternal programming when parents and offspring are in conflict (Paitz et al. 2011), embryos may not rapidly metabolize or convert androgens after they have begun to produce their own endogenous hormones. To determine if the timing of steroid elevation matters, we also injected eggs on Day 6 of incubation, the approximate time of gonadal development in quail embryos (Ottinger and Abdelnabii 1997), in a factorial design producing four treatment groups for the Day 0 and Day 6 treatment combinations: oil‐oil, oil‐hormone, hormone‐oil, hormone‐hormone. The additional (Day 6) injection allowed us to assess whether embryonic growth variation is more sensitive to early elevated androgen exposure (Day 0) or later exposure (after Day 6), regardless of the possibility for rapid metabolism and/or conversion following both injections. Since we did not have published or preliminary data to assess embryonic production of steroids at Day 6, we used the same concentration of A4 + T for both rounds of injections.

We measured multiple body parts (limbs, beak, eyes, mass) of embryos on days 6 and 15 of incubation to assess overall bodily growth in a multivariate analysis, given the possibility of androgens effects on skeletal growth and mass in developing birds (Navara et al. 2005). In addition to body size measurements, we measured embryonic heart rate on days 9, 12, and 15 of incubation as heart rate can be a noninvasive index of developmental rate and embryonic metabolic expenditure (Du et al. 2010; Sheldon et al. 2018; McClelland et al. 2021), providing an additional metric of embryonic development even if embryos did not eventually survive to the final sampling date. Because androgen effects may be dependent on the sex of the embryos (e.g., Henry and Burke 1999), we determined and included embryo chromosomal sex in our analyses.

We hypothesized that (1) androgen injections would increase the overall growth rate of the quail embryos regardless of injection time, (2) androgen injections would increase embryonic metabolic expenditure (heart rate), (3) later injections would have a greater impact on growth rate than earlier injections if early‐injected hormones were converted and/or metabolized more rapidly than the endogenous‐mimicking later hormone injections, and/or (4) hormone injections would increase the concentrations of some other hormones present in the yolk due to the conversion of the androgens, including etiocholanolone and estrogens.

## 2 Materials and Methods

We obtained fertilized wildtype Japanese Quail eggs (*N* = 122 total) from a commercial breeding farm (Purely Poultry, Fremont WI, USA), measured the length and width of each egg (to the nearest 0.1 mm using calipers) and fresh mass of the unincubated egg (to 0.01 g), and froze six eggs at −20°C before incubation to measure initial hormone concentrations. At the time of our study (2019–2020), these experiments were deemed exempt from protocol approval by the University of Illinois Urbana‐Champaign's IACUC.

**Figure 1:**
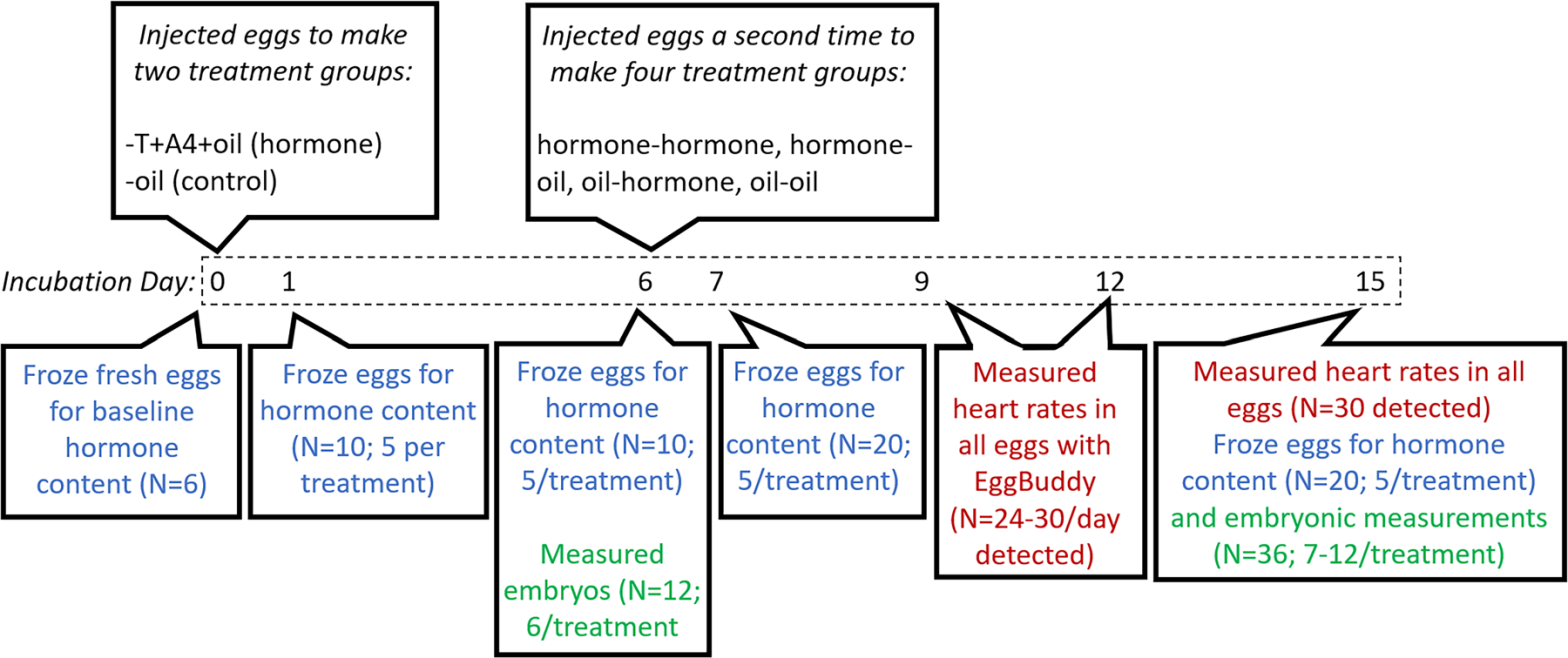
We injected biologically relevant concentrations of testosterone and androstenedione steroid hormones (two standard deviations above average levels) into fertile Japanese Quail (*Coturnix japonica*) eggs before incubation (Day 0) and again 6 days into incubation. We froze eggs for hormone analyses (Days 0, 1, 6, 7, and 15), measured heart rates of embryos (Days 9, 12, and 15), and sampled eggs for embryonic size analyses (Days 6 and 15).

We randomly assigned eggs to one of four treatments (Figure 1): oil‐oil (oil control injection on both day 0 and 6, *N* = 28 eggs), oil‐hormone (oil control on day 0, A4 and T cocktail on Day 6, *N* = 27 eggs), hormone‐oil (A4 and T on day 0, oil on day 6, *N* = 32 eggs), or hormone‐hormone (hormone injections on both day 0 and 6, *N* = 29 eggs) and gave them individual labels with a soft‐tipped semipermanent marker (Sharpie^TM^Newell Brands, Atlanta Georgia, USA). Powdered testosterone (4‐Androsten‐17β‐OL‐3‐one, Steraloids Inc., Newport RI USA) and androstenedione (4‐Androsten‐3,17‐dione, Steraloids Inc., Newport RI USA) were gently agitated in canola oil until dissolved, creating a mixture with a concentration of 140 ng of A4 and 26 ng of T per 10 µL of oil. Using a small sterile needle, we pierced a hole in the eggshell approximately 1 cm below the pointed top of the egg, injected 10 µL of either canola oil (“oil”) or this A4 + T hormone cocktail into the albumen of the egg using a sterile micropipette tip. Previous research indicates that this hormone‐containing oil bolus would float near the hydrophobic yolk (von Engelhardt et al. 2009) and that albumen‐injected steroid hormones can also be recovered in the yolk and the embryo at comparable recovery rates to yolk‐injected hormones (Paitz and Cagney 2019; Paitz et al. 2020). We then re‐sealed the eggshell with a permanent glue adhesive (Krazy^TM^ Glue, Elmer's Products Inc., Westerville, OH, USA). We chose these concentrations because they represent two standard deviations above the average levels of naturally occurring A4 and T concentrations in *Coturnix* eggs (R. Paitz, unpublished data).

After the initial injections we randomly assigned eggs to one of three incubators (Maxi II Eco manual 30 egg incubator, Brinsea Products Inc., Titusville FL, USA) set to incubate the eggs at 37.5°C and 45% humidity and turn the eggs 60° every 40 min. Each incubator was placed under an individual wooden box to control light; the incubators were lit with natural‐light LEDs (Minger, Govee Moments, Hong Kong, HK) for 2 h a day throughout incubation.

Twenty‐four hours after the onset of incubation (Day 1 of ~17‐day incubation) we removed five eggs that had been injected with oil on Day 0 and five eggs that had been injected with the hormone cocktail on Day 0 and froze them for Day 1 hormone analysis (*N* = 10 total, five per treatment). After 6 days of incubation (Day 6) we froze an additional five eggs from the Day 0 oil injection group and five eggs from the Day 0 hormone cocktail injection group for Day 6 hormone analysis (*N* = 10 total, five per treatment). To measure embryo development and because 6‐day‐old embryos' fragile limbs can be damaged by freezing, on Day 6 we also dissected six fresh (unfrozen) eggs from the initial (Day 0) oil injection group and six eggs from the initial (Day 0) hormone injection group (*N* = 12 total embryo measurements), removing the embryo from the yolk sac and measuring its bill, outstretched wing and leg length, and eye diameter down to 0.1 mm with calipers. After 6 days of incubation, it was unclear if eggs without visible embryos were unfertilized eggs or fertile eggs with embryos that died after injection (before visible cell proliferation), so we classified eggs as “living” as the embryo was fully formed and had no signs of decay, and as “unviable/dead” if the embryo had signs of decay or was not present. We measured the embryos' mass to the nearest 0.01 g.

On Day 6 we injected the remaining eggs (*N* = 85) with the second (Day 6) oil or hormone cocktail injection in the factorial design previously described and returned them to the incubator. Twenty‐four hours after the second (Day 6) injections, we froze five eggs from each of the four treatments for Day 7 hormone analyses (*N* = 20 eggs, 17 had viable embryos, with 4–5 per treatment group).

On Days 9, 12, and 15 of incubation we used modified noninvasive ballistocardiogram monitors (EggBuddy, Avitronics, Cornwall, UK) to measure the heart rates of the embryos in all remaining eggs (*N* = 24–30 heart rates detected per day) and recorded data in real‐time (Di Giovanni et al. 2023). We placed each egg into the monitor at ambient room temperature and recorded 5 min of activity. In Program R (R Core Team 2013) we calculated the average and maximum heart rate measured over those 5 min.

On Day 15 we froze an additional five eggs from each of the four treatments for hormone analyses (*N* = 20 eggs) and dissected the remaining eggs, measuring the embryos' bill, wing, and tarsus length, eye diameter, and mass. Due to inviable eggs and embryo mortality, we also measured the embryos in the eggs frozen for hormone analyses (stored at −20°C for 35 days), leaving us with nine embryos on Day 15 for oil‐oil, seven for treatment oil‐hormone, eight for hormone‐oil, and 12 for hormone‐hormone. All experimentation was approved by the University of Illinois IACUC as exempt because avian embryos were not considered live animals by U.S. regulatory agencies and institutional guidelines for avian embryos were not revised until 03 January 2023 (after this experiment was completed). All animal handling was done in accordance with the National Research Council's Guide for the Care and Use of Laboratory Animals.

We prepared yolk samples (*N* = 63 total, 6–20 per sampling day) and extracted steroids using methods of Merrill et al. (2019), quantifying the concentrations of 29 yolk steroid hormones in ng/g. Targeted hormones included the *estrogens* estrone and estradiol; *androgens* androstenedione, testosterone, dehydroepiandrosterone (DHEA), etiocholanolone, and 11‐ketotestosterone; *glucocorticoids* 11‐dexoycortisol, cortisone, 5β‐dihydrocortisone, 20β‐dihydrocortisone, cortisol, 5β‐dihydrocortisol, 5β‐tetrahydrocortisone, 5β‐tetrahydrocortisol, β‐cortol, corticosterone, 5β‐tetrahydrocorticosterone, 11‐dehydro‐tetrahydrocorticosterone, 5β‐corticosterone, deoxycorticosterone, 11α‐hydroxyprogesterone, and 20β‐dihydrocorticosterone; and *progestogens* progesterone, pregnenolone, 17α‐hydroxypregnenolone, dihydroprogesterone (DHP), pregnanedione, and pregnanolone. We removed yolks from frozen eggs, homogenized the yolks, mixed 0.5 g of homogenized yolk in 4 mL 100% methanol, and stored them at −20°C overnight. We centrifuged the samples for 15 min at 200 r/min at 4°C, extracted the supernatant and added 46 mL deionized water to dilute it for solid phase extraction of steroids. We loaded the supernatant into C18 Sep‐pak cartridges (Waters Ltd., Watford, UK) charged with 4 mL 100% ethanol and rinsed with water, performing solid‐phase extraction under vacuum pressure (1.5–2.5 psi). We rinsed the filters with water before eluting the samples with 5 mL ether, drying them overnight, and submitting them to the Roy J. Carver Biotechnology Center's (University of Illinois at Urbana‐Champaign) Metabolomics Laboratory for LC‐MS‐MS analysis on a 5500 QTrap LC‐MS‐MS system (AB Sciex, Foster City, California, USA). Hormones were detected at a ~ 10 pg threshold (Merrill et al. 2019; Hauber et al. 2020) and we calculated percent recovery with two yolk samples spiked with a standard solution that contained 1000 pg of the sample hormones. Recoveries ranged from 56% for 17OH‐hydroxypregnenolone to 82% for pregnenedione.

To test for sex‐specific effects on development we extracted DNA from each embryo's tissue using Qiagen DNeasy Blood and Tissue Kits, following the standard extraction protocol (Qiagen Inc., Germantown MD, USA). We amplified DNA using ASM‐PCR at the Chromodomain Helicase DNA binding 1 (CHD1) gene with three primers (Coustham et al. 2017): ZF (5ʹ‐CTCTGGGTTTTGACTGTATTG‐3ʹ), WF (5ʹ‐CATCTGTTTTCCCCCCCAAA‐3ʹ), and P2 (5ʹ‐TCTGCATCGCTAAATCCTTT‐3ʹ). We mixed 1 µl DNA, 0.67 µM of each ZF and WF primers, 1.4 µM of P2 primers, and Thermo Scientific DreamTaq Green Master Mix (Thermo Fisher Scientific, Waltham, MA, USA) for a total reaction volume of 15 µL. PCR was performed at 95°C for 2 min, followed by 30 cycles at 95°C (30 s), 52°C (45 s) and 72°C (60 s) and a final extension at 72°C for 5 min run on a Bio‐Rad T‐100 Thermocycler (Bio‐Rad Industries, Hercules, CA, USA). We visualized 10 µL of the PCR product and PCR product from known‐sex positive controls on a 1% agarose gel stained with GelRed (Biotium, Fremont CA, USA), identifying female embryos by two bands and male embryos by one band (Coustham et al. 2017).

We used linear models in Program R (R Core Team 2013) to determine whether the eggs assigned to each treatment varied in their initial mass, length, or width. We compared the survival of the embryos (the number of living embryos compared to eggs with no visible embryo and eggs with a dead embryo) to Day 6 and Day 15 with chi‐squared analyses. We performed multivariate analysis of variance (MANOVA) models, which assess the relationship of predictor(s) on several response variables that may be correlated, to model predicted treatment effects on overall embryonic size. With separate MANOVAs, we assessed the effect of incubator assignment, initial (Day 0) hormone injection, embryo sex, and injection treatment × sex on the leg length, bill length, wing length, eye diameter, and mass of embryos on Day 6 of incubation. Similarly, we used MANOVAs to investigate the effect of incubator assignment, initial (Day 0) hormone injection, second (Day 6) hormone injection, two‐step (Day 0 and Day 6) hormone injection, sex, and hormone × sex interactions on the tarsus length, bill length, wing length, eye diameter, and mass of the embryos on Day 15 of incubation. We present effect sizes as eta‐squared values with 95% confidence intervals. When these MANOVA results were significant at α = 0.05 we performed a linear discriminant analysis (LDA) with the same predictors as the MANOVA. These LDAs allowed us to explore the response variables that varied most between the treatment groups and examine the percent contributions of the predictor(s) to observed differences in embryonic size. We used LDAs rather than a series of univariate analysis of variance (ANOVA) models to examine drivers of multivariate differences while minimizing the number of statistical comparisons. LDAs were performed with the R package *MASS* (Venables and Ripley 2002).

We used linear mixed models with embryo ID as a random effect to assess the effect of sampling day, incubator assignment, and hormone treatment on average embryo heart rate, analyzing treatment effects with a Type III ANOVA. Since we had heart rate data for all eggs but sex data for only the embryos in eggs dissected for morphological measurements (not the eggs sampled for hormone concentrations), we ran the same models of embryo heart rate, with the addition of sex as a predictor, for only the subset of eggs for which we had embryo sex data.

We compared the initial concentrations of detected hormones in Day 0 eggs between treatment groups with linear models, including investigating possible natural correlations between T and A4 using a Pearson's correlation analysis. To determine whether the injections elevated T and A4 concentrations in the yolks of treated eggs we compared concentrations of eggs injected with oil to those injected with the hormone cocktail 24 h after injection (Day 1 following the Day 0 injection, Day 7 following the Day 6 injection) using linear models. We used MANOVAs to compare hormone profiles across incubators, sampling days, between treatment groups, and treatment × sampling day interaction.

Since we measured embryos and hormone concentrations simultaneously in a subset of eggs (*N* = 20) collected on Day 15 we were able to investigate direct relationships between embryonic size and yolk hormone concentrations in these eggs. We performed separate principal component analyses based on embryo size metrics on Day 15 (Table S1) and yolk hormone profiles from the same eggs on Day 15 (Table S2). We assessed the effects of hormone principal components (PCs), hormone injection treatment, hormone PC × injection treatment, embryo sex, and hormone PC × sex on embryo size principal components using linear models. We also assessed the impact of embryo sex and an embryo sex × injection treatment interaction on the hormone concentration PCs using linear models.

## 3 Results

Eggs randomly assigned to each treatment did not vary in initial mass (mean ± SE = 22.33 ± 10.56 g, *F*_3,118_ = 0.93, *p* = 0.430), length (mean ± SE = 35.35 ± 0.10 mm, *F*_3,118_ = 0.83, *p* = 0.478), or width (mean ± SE = 27.75 ± 0.07 mm, *F*_3,118_ = 1.07, *p* = 0.367). On Day 6 95.7% of the sampled eggs (*N* = 22 of 23 eggs) contained a living embryo (with one sampled egg unfertilized/undeveloped) and embryo survival was not impacted by hormone injection treatment (χ^2^_1_ < 0.001, *p* > 0.999). Likewise, on Day 15 65.5% of the sampled eggs (*N* = 38) contained a living embryo, with the remaining eggs unfertilized/undeveloped (*N* = 10) or containing a dead embryo (*N* = 17) and embryo survival was not affected by hormone injection treatment (χ^2^_3_ = 0.72, *p* = 0.870, with *N* = 4–5 dead per treatment).

### 3.1 Embryo Sizes and Sex Differences

**Figure 2:**
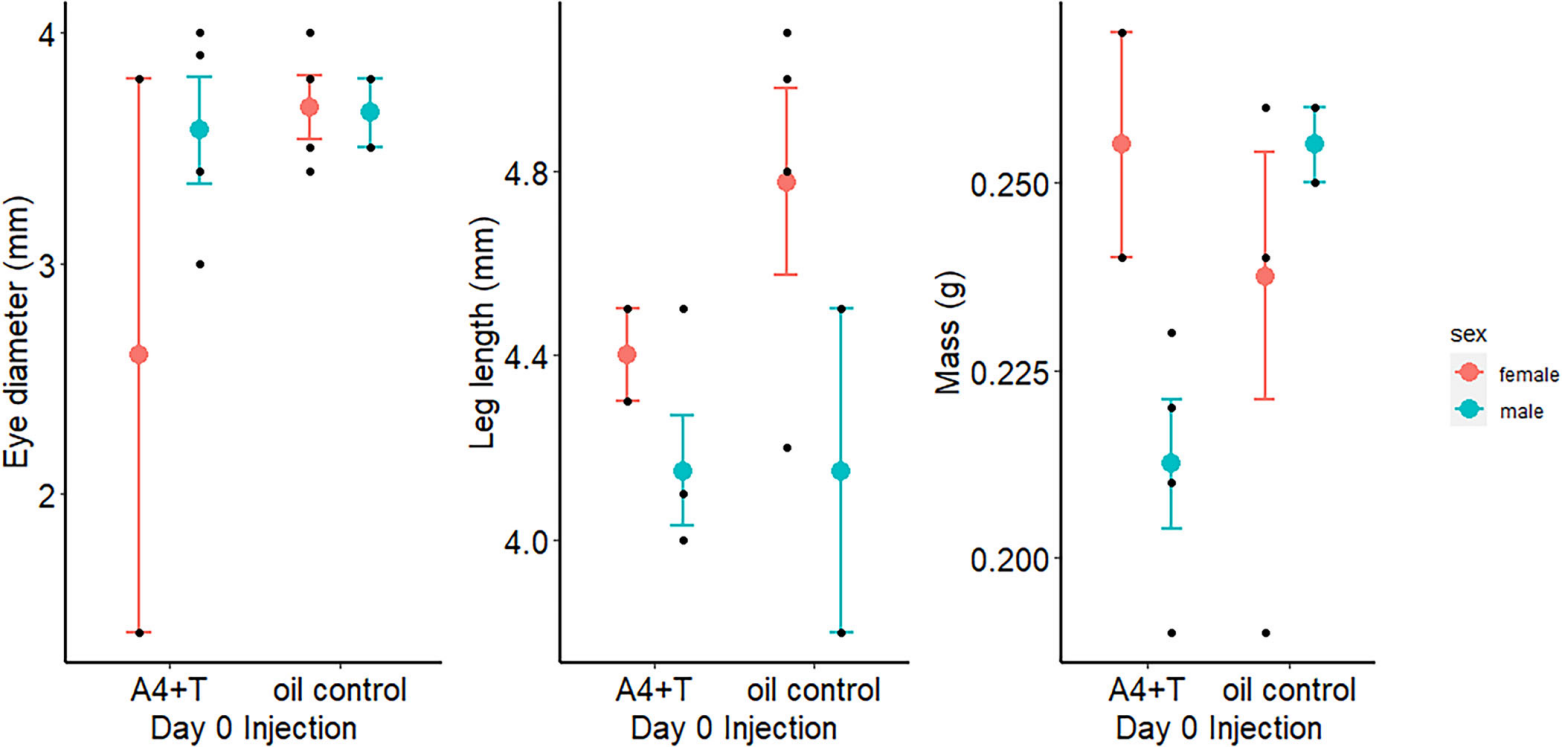
After 6 days of incubation, overall Japanese Quail (*Coturnix japonica*) embryo size was affected by an interaction between embryonic sex and the A4 + T hormone injection administered to eggs on Day 0. Post‐hoc analyses (LDAs) indicated that this multivariate response was primarily driven by embryonic eye diameter, leg length, and mass; the raw data are presented here (black points). Female embryos in eggs injected with the androgen hormones had smaller eye diameters (mm) than male embryos injected with hormones or embryos of either sex injected with the oil control (left). Male embryos had smaller leg lengths (mm) than female embryos regardless of injection treatment, while females in eggs injected with hormones had shorter legs than females injected with oil (center). Male embryos in eggs injected with hormones had lower body mass measurements (g) than female embryos and male embryos in eggs injected with the oil control (right). Colored points indicate the mean and standard errors.

On Day 6, embryo size (*N* = 12 embryos, two treatments) was affected by an interaction between hormone injection treatment and embryo sex (η^2^[95%] = 0.91 [0.27–1.00], *F*_5,4_ = 8.05, *p* = 0.033). A linear discriminant analysis of the effect of Day 0 hormone injection × embryonic sex produced two linear discriminant scores with trace values > 10% (Table 1). The first LD score described 82.0% of the variation in the size data (Table 1) and illustrated the hormone injection × sex interaction (Figure S1), with the covariate contributions of each body part measurement to this axis indicating that the embryos varied most in their eye diameters, leg lengths, and body mass measurements (Table 1). Specifically, female embryos injected with A4 + T had smaller eye diameters than female embryos developing in eggs injected with oil or male embryos in either injection treatment (Figure 2). While male embryos in either injection treatment had shorter legs than female embryos, female embryos in eggs injected with A4 + T had shorter legs than females in eggs injected with oil (Figure 2). Male embryos in eggs injected with A4 + T had lower mass measurements than males in eggs injected with oil or female embryos in either injection treatment (Figure 2).

**Table 1:** Linear discriminant (LD) values for the first three linear discriminant analyses of the effect of pre‐incubation (Day 0) A4 + T injection treatment on 6‐day‐old Japanese Quail (*Coturnix japonica*) embryos’ size measurements, with proportion of trace values indicating the amount of variation captured by each of the three analyses and individual values for each body part in the analysis.

	LD1	LD2	LD3
*Proportion of trace:*	*0.820*	*0.166*	*0.015*
**Body part:**			
Leg length (mm)	**−7.443**	−1.938	0.218
Bill length (mm)	3.507	**3.598**	−0.350
Wing length (mm)	−2.432	**−4.584**	0.120
Eye diameter (mm)	**−6.117**	2.731	−2.472
Mass (g)	**8.378**	0.203	−1.478

*Note:* Bold values indicate the largest LD1 and LD2 values.

**Figure 3:**
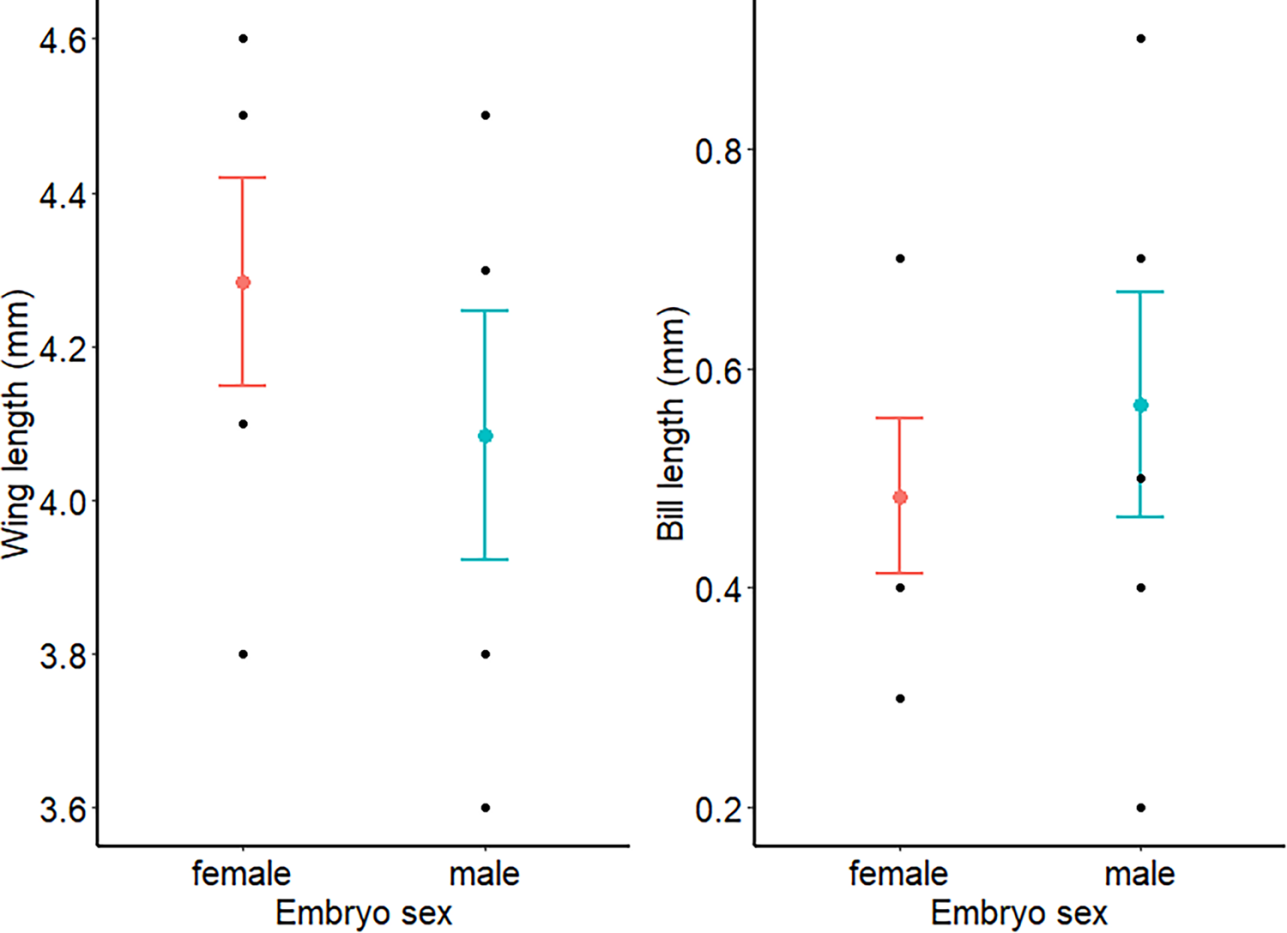
Following 6 days of incubation, female Japanese Quail (*Coturnix japonica*) embryos had larger average wings (in mm, left) and shorter average bills (mm, right) than male embryos. Colored points and brackets indicate the mean and standard error, while black points show the measured data.

The second LD score described 16.6% of the variation in the size data (Table 1) and showed sex differences in body size (Figure S1), with the covariate contributions of each body part measurement to this axis indicating that the sexes varied most in their wing and bill lengths (Table 1). Female embryos' wings were, on average, 4.9% (0.2 mm) longer than male embryos' wings, regardless of Day 0 injection treatment (Figure 3). Male embryos' beaks were, on average, 17% (0.08 mm) longer than female embryos' beaks, regardless of the Day 0 injection treatment (Figure 3). Incubator assignment had no effect on embryo size on Day 6 (η^2^[95%] = 0.56 [0.00–1.00], *F*_10,12_ = 1.54, *p* = 0.237).

**Figure 4:**
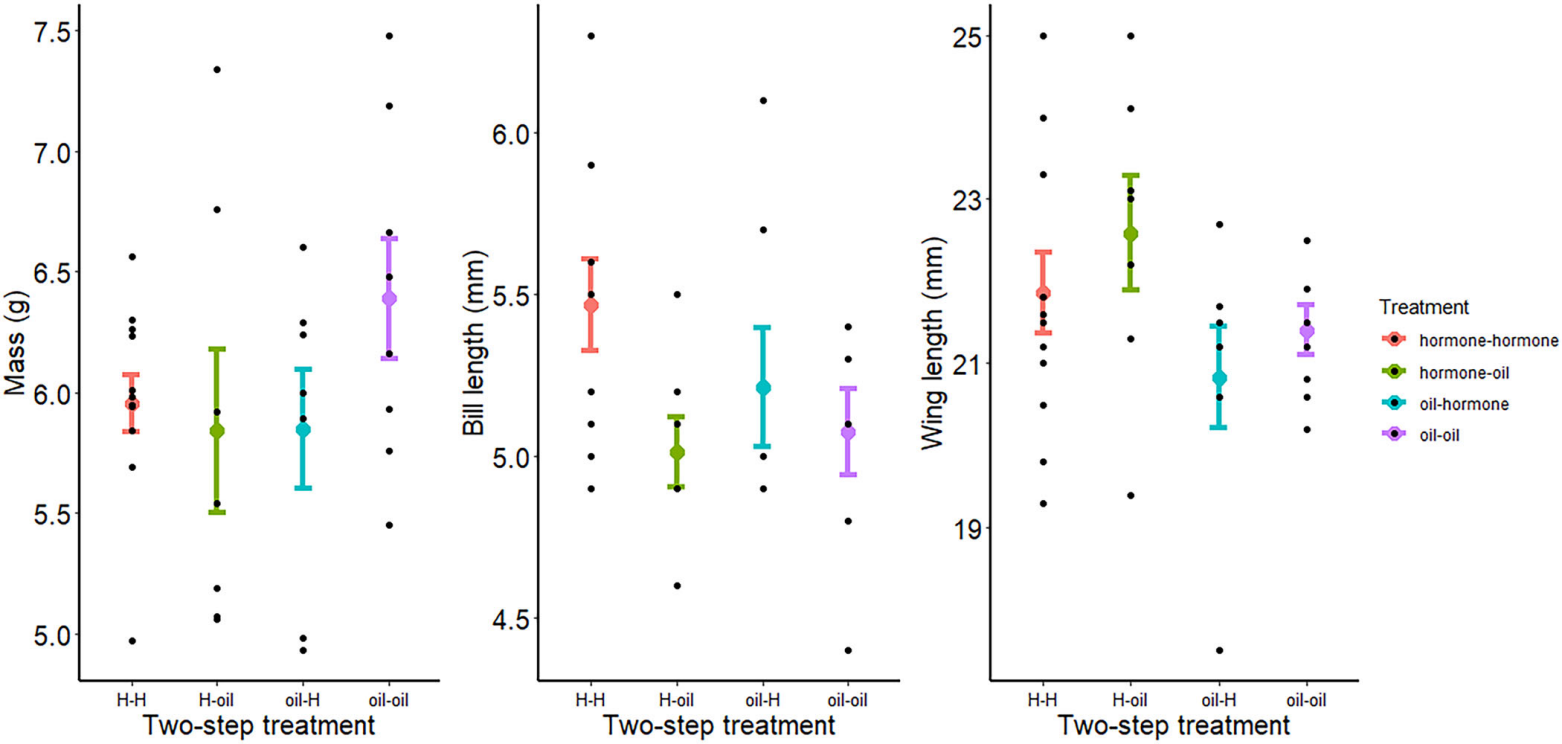
After 15 days of incubation, Japanese Quail (*Coturnix japonica*) embryos developing in eggs injected with either androgen hormones or an oil control (on both Days 0 and 6 of incubation) exhibited variation in their mass (in g; left), bill length (mm; center), and wing length (mm; right). A linear discriminant analysis suggests that the eggs injected with A4 + T hormones on both sampling days (hormone‐hormone, red) produced embryos with a lighter mass and longer beaks and wings than the embryos from eggs injected with an oil control on both days (oil‐oil, purple). Colored points and brackets indicate the mean and standard error, while black points show the measured data.

By Day 15 embryo size (*N* = 36 embryos, four treatment groups) was significantly impacted by the hormone injection administered on Day 0 alone (η^2^[95%] = 0.32 [0.00–1.00], *F*_15,28_ = 2.58, *p* = 0.049) and marginally affected by the second (Day 6) injection alone (η^2^[95%] = 0.31 [0.00–1.00], *F*_15,28_ = 2.52, *p* = 0.053). The combined two‐step (both Day 0 and Day 6 hormone injection) treatment significantly affected embryonic size (η^2^[95%] = 0.28 [0.01–1.00], *F*_15,72_ = 1.85, *p* = 0.043) but the sex × treatment interaction was not significant (η^2^[95%] = 0.19 [0.00–1.00], *F*_15,72_ = 1.09, *p* = 0.378). A linear discriminant analysis of the two‐step (Day 0 and Day 6) injection treatment produced three LD axes with proportion of trace values > 10% (Table 2). The first LD axis described 59.7% of the variability in embryonic size (Table 2) but did not effectively sort the embryos by hormone treatment; the hormone‐hormone treatment (eggs that received A4 + T on both Days 0 and 6) sorted from the oil‐oil treatment, but the hormone‐oil and oil‐hormone points were interspersed (Figure S2). The covariate contributions of each body part measurement to this axis indicated that the embryos varied most in their bill lengths, wing lengths, and mass measurements (Table 2). Eggs injected with hormones on both days (0 and 6) produced embryos that had longer beaks, longer wings, and lighter mass measurements than embryos injected with oil on both days (Table 3, Figure 4). LD2 and LD3 scores did not sort the embryos by treatment (Figure S2). Incubator assignment had no effect on embryo size on Day 15 (η^2^[95%] = 0.09 [0.00–1.00], *F*_10,56_ = 0.53, *p* = 0.860).

**Table 2:** Linear discriminant (LD) values for the first three linear discriminant analyses of the effect of A4 + T two‐step (Day 0 and 6) injection treatment on 15‐day‐old Japanese Quail (*Coturnix japonica*) embryos’ size measurements, with proportion of trace values indicating the amount of variation captured by each of the three analyses and individual values for each body part in the analysis.

	LD1	LD2	LD3
*Proportion of trace:*	0.597	0.222	0.182
**Body part:**			
Tarsus length (mm)	1.402	1.304	2.612
Bill length (mm)	**4.153**	0.092	3.134
Wing length (mm)	**5.164**	−2.793	0.170
Eye diameter (mm)	−2.774	−2.941	1.308
Mass (g)	**−4.114**	0.513	−0.465

*Note:* Bold values indicate the largest LD1 values.

**Table 3:** Mean values and standard deviations for Japanese Quail (*Coturnix japonica*) embryo mass (in grams), bill length (mm), and wing length (mm), following 15 days of incubation.

	Mass (g)	Bill length (mm)	Wing length (mm)
	Mean ± SD	Mean ± SD	Mean ± SD
**Two‐step injection treatment**			
Oil‐oil	6.39 ± 0.70	5.08 ± 0.37	21.40 ± 0.86
Oil‐hormone	5.85 ± 0.65	5.21 ± 0.48	20.83 ± 1.63
Hormone‐oil	5.84 ± 0.90	5.01 ± 0.29	22.59 ± 1.85
Hormone‐hormone	5.95 ± 0.40	5.47 ± 0.49	21.86 ± 1.72

*Note:* Eggs were injected before incubation (Day 0) with androgen hormones or an oil control, and injected again on Day 6 with androgen hormones or an oil control in a factorial design (two‐step injection treatment).

### 3.2 Heart Rates

Average embryo heart rate (*N* = 24–30 embryos/day) was not impacted by sampling day (χ^2^_1_ = 0.40, *p* = 0.527), incubator assignment (χ^2^_2_ = 1.04, *p* = 0.595), or injection treatment (χ^2^_3_ = 5.16, *p* = 0.160, Figure S3). Adding sex to the same model (with a subset of data that includes sexed embryos) did not better explain the average embryo heartrate (sex additive effect: χ^2^_1_ = 0.09, *p* = 0.767).

### 3.3 Yolk Steroid Hormone Concentrations

We detected 11 of the 29 targeted hormones on Day 0 before incubation (Table S3), including pregnanolone, pregnanedione, 17α‐hydroxypregnenolone, 17α‐hydroxyprogesterone, progesterone, pregnenolone, 11‐ketotestosterone, etiocholanolone, dehydroepiandrosterone (DHEA), testosterone (T), and androstenedione (A4). In the unincubated eggs (*N* = 6 eggs, no treatments), yolk T and A4 were positively correlated (Pearson's r: 0.827, t_4_ = 2.95, *p* = 0.042). The same 11 detected hormones were present on Day 1 after the injection of oil or an A4 + T mixture (*N* = 10 eggs, two treatments, Table S3). Despite the injections of A4 and T into half of the eggs, the treatment groups did not differ in their yolk concentrations of A4 (*F*_1,8_ = 1.86, *p* = 0.210, Figure S4) or T the day after injections (*F*_1,8_ = 0.65, *p* = 0.445, Figure S4).

By Day 6 of incubation (*N* = 10 eggs, two treatments), we no longer detected 17α‐hydroxyprogesterone or 11‐ketotestosterone in the eggs (Table S4). On Day 7, one egg in the hormone‐hormone treatment had detectable levels of 5β ‐tetrahydrocortisol in addition to the nine hormones detected in the rest of the eggs (Table S5). Following the second injection of A4 and T (*N* = 17 eggs, 4–5 for each of four treatments), eggs injected with these hormones showed no significant difference in A4 (*F*_1,13_ = 1.15, *p* = 0.301, Figure S5), or T (*F*_1,15_ = 3.35, *p* = 0.087, Figure S5) levels relative to eggs injected with oil.

**Figure 5:**
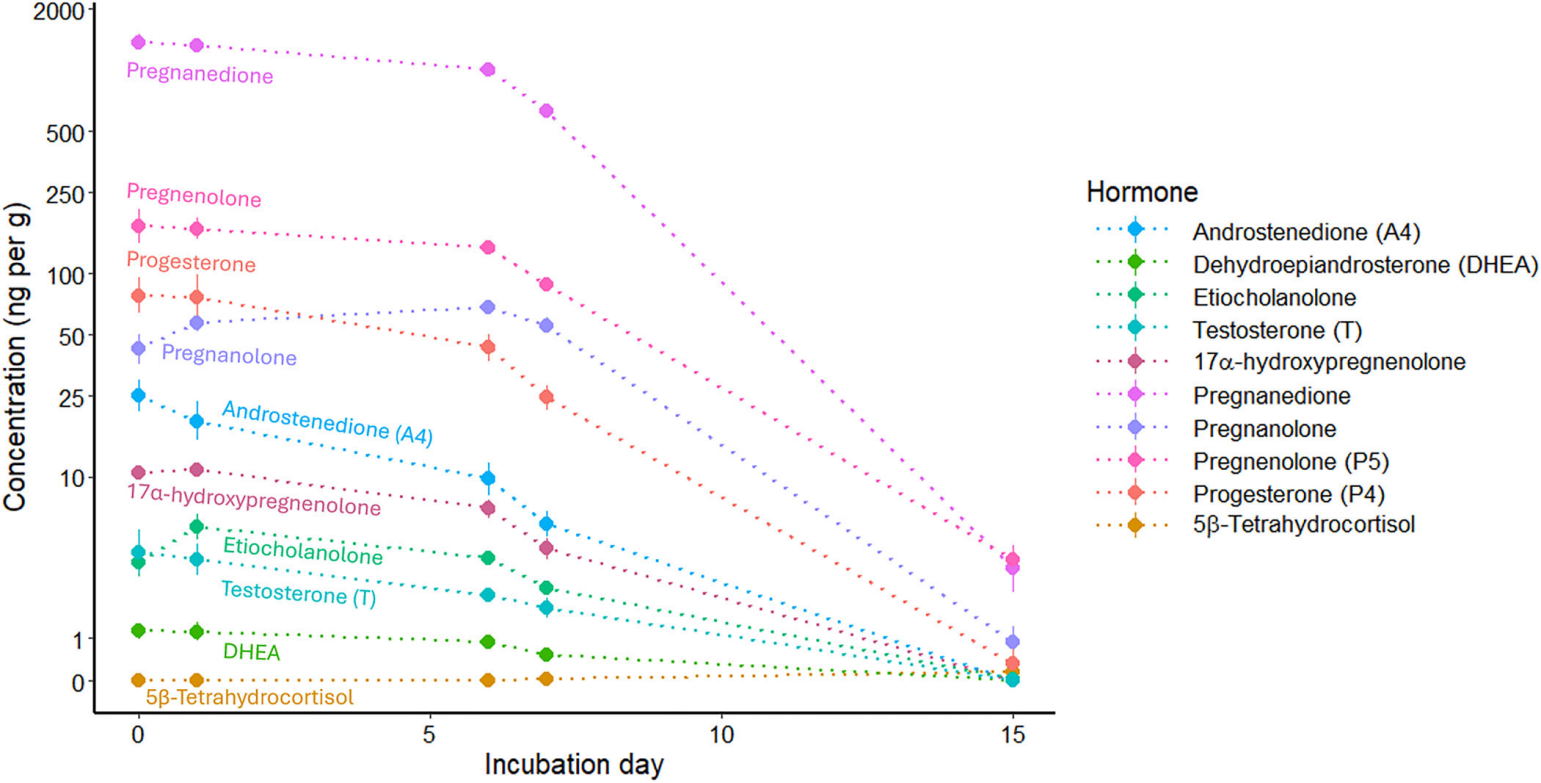
The concentration (ng/g) of 10 steroid hormones detected across all Japanese Quail (*Coturnix japonica*) egg yolks in the experiment declined over the sampling days (days 0, 1, 6, 7, and 15 of incubation; *F*_12,44_ = 45.62, *p* < 0.001). Points indicate the mean values at each sampling day with vertical standard errors. Sample sizes varied by sampling point (Day 0 N = 6, Day 1 N = 10, Day 6 N = 10, Day 7 N = 17, and Day 15 N = 20). Note that the concentrations on the y‐axis are log‐scaled and that the dotted lines connecting points do not indicate measured values of the same individuals but simply connect means measured for different sub‐sets on days 0, 1, 6, 7, and 15 of incubation.

On Day 15 (*N* = 20 eggs, five for each of four treatments) we only detected 6 hormones in any egg, including progesterone, pregnenolone, pregnanedione, and pregnanolone in all eggs, 5β‐tetrahydrocortisol in four eggs, and etiocholanolone in one egg (Table S6). Hormone concentrations in the yolk decreased with sampling day (η^2^[95%] = 0.92 [0.89–1.00], *F*_10,46_ = 56.13, *p* < 0.001, Figure 5), but the hormone treatment × sampling day effect was not significant (η^2^[95%] = 0.22 [0.00–1.00], *F*_30,144_ = 1.35, *p* = 0.125). Incubator assignment did not impact yolk hormone concentrations (η^2^[95%] = 0.22 [0.00–1.00], *F*_20,72_ = 0.99, *p* = 0.480).

### 3.4 Day 15: Embryo Size PCA and Yolk Hormone PCA

On Day 15 we had 20 eggs with simultaneous hormone and embryo size measurements (*N* = 5 for each of the four treatments), allowing us to compare embryo size to remaining hormones in the yolk. The first and second principal components of hormone concentrations (hormone PC1 and hormone PC2, Table S1) did not explain the first embryo PC (effect of hormone PC1: β ± SE: −2.12 ± 1.51, *t*_18_ = −1.40, *p* = 0.178; effect of hormone PC2: β ± SE: −0.05 ± 0.18, *t*_18_ = −0.27, *p* = 0.794, Table S7) or the second embryo PC (effect of hormone PC1: β ± SE: 0.473 ± 1.19, *t*_18_ = 0.40, *p* = 0.696; effect of hormone PC2: β ± SE: −0.24 ± 0.24, *t*_18_ = −1.02, *p* = 0.323, Table S7). An additional hormone injection treatment term and an injection × hormone principal component interaction term were not significant in any model (*p* = 0.081–0.963, Table S7), nor were the effects of sex and a sex × hormone principal component interactions (*p* = 0.134–0.964, Table S7). Embryonic sex had no statistical relationship with the hormone concentration principal components on Day 15 (*p* = 0.261–0.544, Table S7). The interaction between embryonic sex and the hormone injection treatment similarly had no relationship with the yolk hormone concentration principal components (*p* = 0.237–0.367, Table S7).

## 4 Discussion

Injecting biologically relevant quantities of A4 + T (adding two standard deviations above the average concentration) simultaneously into unincubated quail eggs and again into eggs 6 days into development did not produce markedly larger quail embryos as we had hypothesized. Instead, androgen hormone exposure appeared to temporarily reduce early embryonic growth in sex‐dependent ways. Six days after the initial hormone injection, male embryos exposed to the hormone injection were lighter than male embryos exposed to the oil control, while female embryos exposed to the hormone injections had smaller eye diameters and leg lengths than female embryos exposed to the oil control. However, by Day 15 of incubation ( ~ 2 days before natural hatch dates), there was no sex‐dependent effect of hormone injection treatment on embryonic size. The embryos in eggs subjected to two doses of A4 + T (on Days 0 and 6) had slightly longer beaks and wings but lower mass measurements than the embryos in eggs that received the oil control on both Days 0 and 6, whereas the oil‐hormone and hormone‐oil treatments did not produce embryos with significant size differences. While these results are based on a small sample size for each sex × injection treatment combination, and should therefore be replicated in future studies, they suggest that initial (Day 0) A4 + T injections may have reduced early‐development growth rates in a sex‐dependent manner, but these differences diminish close to hatch times unless the embryos received two doses of A4 + T or are simply undetectable given our sample sizes.

Maternally derived A4 + T naturally co‐occur in avian egg yolks and concentrations are often correlated (Hegyi et al. 2011) as they were in our initial (pre‐incubation) yolk measurements, suggesting that research into the role of these hormones in embryonic development should investigate effects of these androgens in tandem. Our results add to the emerging patterns (Sockman and Schwabl 2000; Eising and Groothuis 2003; Müller and Eens 2009) indicating that, when increased simultaneously, A4 and T can influence embryonic development, but not by simply altering overall growth rates. If elevating these androgens in tandem does speed or slow growth rates in quail, it appears to occur in a sex‐dependent and structure‐dependent manner. Prior studies of steroid concentrations in blood serum showed that avian embryos' exposure to steroids varied by sex at later stages of development (Wang et al. 2019), as did their own production of steroid‐associated enzymes (Bruggeman et al. 2002) and tissue‐specific gene expression (Smith and Sinclair 2001). Because sex‐dependent growth studies have not focused on limbs or eyes, it is currently unknown if quail have sex‐dependent steroid target tissues in these regions of their body. We had few individual embryos for each sex in each treatment combination, especially on Day 6 of incubation when we only had two female embryos in the hormone‐exposed treatment and two male embryos in the oil‐exposed control. Future studies of early sex‐dependent growth should sacrifice more eggs at each time point while studies of sex‐dependent growth later in development should consider assessing sex before eggs are selected for sampling (i.e., through blood serum measurements, Wang et al. 2019) to ensure that the results are not driven by a bias in the sex of the randomly‐selected embryos.

Our results, showing temporary and sex‐dependent variation in growth with androgen injection, stand in some contrast to previous studies showing consistent increased skeletal growth with increased androgen exposure (e.g., Schwabl 1996b; Benowitz‐Fredericks and Hodge 2013; Muriel et al. 2013), and sex‐specific effects of androgens on size (Tschirren 2015) and immune responses (Muriel et al. 2017). However, it is important to note that those studies assessed hatchling size and post‐natal growth; steroids could alter post‐hatch behavior to impact post‐hatch growth in ways our study of embryonic growth did not assess (Schwabl 1996b). Even if the impacts of steroid exposure on embryonic growth are short‐lived, such variation in growth rate may result in life‐long variation in size which in turn impacts fitness, given that it may influence survival to adulthood (Remeŝ and Martin 2002), although in our study exposure to hormone injections did not influence embryonic survival to Day 15 ( ~ 2 days before average hatch date). Future work on these hormones should connect these small differences in embryonic development to hatch time and success, size of the birds at hatch, and their fitness following hatch to truly inform our understanding of how correlated egg yolk androgens can influence developing birds throughout their life.

Previous hormone‐injection studies (Adkins 1979; Ottinger et al. 2001) have indicated that the timing of androgen exposure can influence the phenotypes of developing quail. When we injected A4 and T later in development (Day 6), we found no significant impact of that injection on the size of embryos, suggesting there may be a critical window in early development (before Day 6) where embryos are most sensitive to yolk steroids. Gonadal function in quail begins at approximately Day 5–6 of incubation (Ottinger and Abdelnabi 1997), activity of androgen metabolizing 5β‐reductase enzymes in embryonic brains does not increase until Days 7–15 of incubation (Balthazart and Ottinger 1984), and the hypothalamus‐pituitary‐gonadal (HPG) endocrine feedback axis does not become active until around days 10–15 of incubation (Li et al. 1991). Therefore, early sensitivity to yolk androgens may be due to androgen receptors in extraembryonic membranes (present by day 5 of incubation in chickens, Kumar et al. 2019). Taken in combination with the initial (Day 0) hormone injection, though, a second hormone exposure may have increased beak length and wing length while decreasing mass relative to the oil‐oil treatment, but the embryos which received hormones on Day 0 had body size measurements that fell between those two averages regardless of whether they received oil or hormones on Day 6.

Androgens can influence growth and development by binding to receptors to influence cellular processes (Falkenstein et al. 2000). Given that embryonic birds have androgen receptors in their tissue early in development (Godsave et al. 2002), it is possible for early androgen exposure to influence processes such as cellular metabolism; while we did not measure metabolic rates directly, our data indicate that artificially elevating A4 and T at two different points in incubation did not influence embryonic heart rate later in development. In addition to these receptors in tissues, embryos may be influenced by the action of androgen receptors in extra‐embryonic membranes, such as the sac surrounding the yolk (Albergotti et al. 2009); the presence and action of these extra‐embryonic androgen receptors may even be influenced by the concentration of androgens present in early development (Kumar et al. 2019). This study did not measure these extra‐embryonic receptors directly, but it is interesting to note that 24 h after both injections (Days 0 and 6) we did not detect measurable increases in T or A4 in the yolks of eggs injected with these steroids, suggesting that our experimental manipulation may not have resulted in increased androgen exposure in the inner yolk layers but rather to the outer yolk membrane.

In addition to this direct‐binding mechanism of action, androgens may be rapidly converted into other active hormones and even rendered into conjugated by‐products with no known mechanism of action (Paitz et al. 2011; Vassallo et al. 2014), even within the first 24 h of incubation in viable eggs (von Engelhardt et al. 2009). Embryos' capacity to metabolize and/or convert hormones may even vary in ecologically relevant ways, such as with the order in which their egg was laid and the corresponding competitive environment they would experience upon hatch in passerines (Kumar et al. 2018). Given that androgens can be aromatized into estrogens but may more often be converted to etiocholanolone (Campbell et al. 2020) we measured the concentrations of estrogens, etiocholanolone, and other steroid hormones in the egg yolk over development. The concentrations of the yolk hormones fell over the course of development in all eggs and treatments. However, we did not find significant differences in the yolk concentration of any of these hormones between the eggs treated with oil controls or androgen hormone injections. Embryo size on Day 15 and embryonic sex was not correlated with the concentrations of hormones in the remaining yolk. Overall, these results suggest that the injected androgens were not being converted into the measured hormones and stored in the yolk, but that does not mean that they were not being conjugated or metabolized into other products with immediate or future biological implications for the developing embryo.

Maternally derived A4 and T naturally occur in bird egg yolks; manipulation of these hormones separately can produce measurable effects on embryonic growth, hatching, nestling development post‐hatch, behavior, and survival (Groothuis et al. 2019). Maternal androgens, therefore, could provide a mechanism of maternal effects, allowing the maternal body to influence the growth and development of offspring and adjust to prevailing environmental and social conditions. These hormones do not act alone, however; their concentrations are often correlated, yet the result of simultaneous elevation of these hormones are not clear. While our study was limited by its small sample sizes, we were able to show that injecting both A4 and T simultaneously influences the early growth of quail embryos in sex‐ and structure‐dependent ways, even though the androgen injections did not elevate the concentrations of any steroid hormones stored in the yolk immediately (24 h) after injection or through 15 days of incubation. It remains to be seen, however, whether these small and possibly temporary differences in embryo size correspond to lasting consequences for the developing embryos' hatching, post‐hatch growth, behavior, adult size, and survival.

## Conflicts of Interest

The authors declare no conflicts of interest.

## Supporting information


**Table S1:** Eigenvalues, percentage of variance explained, and loadings of body parts onto the dimensions of the Day 15 Japanese Quail (*Coturnix japonica*) embryo size principal component analysis. **Table S2:** Eigenvalues, percentage of variance, and loadings of egg yolk hormone concentrations (ng/g) onto the dimensions of the Japanese Quail (*Coturnix japonica*) incubation day 15 hormone principal component analysis. **Table S3:** Yolk steroid hormones present on sampling days 0 and 1 of incubation. Eggs were injected on Day 0 with either oil or +2 SD A4 and T. Means, standard deviations, and min‐max range all ng/gram of yolk. **Table S4:** Yolk steroid hormones present on sampling day 6 of incubation. Eggs were injected on Day 0 with either oil or +2 SD A4 and T. Means, standard deviations, and min‐max range all ng/gram of yolk. **Table S5:** Yolk steroid hormones present on sampling day 7 of incubation. Eggs were injected on Day 0 and Day 6 with either oil or +2 SD A4 and T. Means, standard deviations, and min‐max range all ng/gram of yolk. **Table S6:** Yolk steroid hormones present on sampling day 15 of incubation. Eggs were injected on Day 0 and Day 6 with either oil or +2 SD A4 and T. Means, standard deviations, and min‐max range all ng/gram of yolk. **Table S7:** Statistical models of Japanese Quail (*Coturnix japonica*) embryo body size principal components, egg yolk steroid hormone principal components, injection treatment (Day 0 and Day 6 hormone or oil injections), and embryo chromosomal sex on Day 15 of incubation. **Figure S1:** Linear discriminant (LD) scores illustrate an interaction between the effect of embryonic sex and androgen hormone injection on the size of 6‐day old Japanese Quail (*Coturnix japonica*) embryos. LD1 (x‐axis) scores define a sex × treatment interaction, while LD2 (y‐axis) scores show a sex effect on body size. **Figure S2:** Linear discriminant (LD) scores for LD 1 (x‐axis, both plots), 2 (y‐axis, left), and 3 (y‐axis, right) show little separation in Japanese Quail (*Coturnix japonica*) embryonic body size measurements on Day 15 of incubation following androgen hormone or oil control egg injections (on both Days 0 and 6 of incubation). LD1 scores, which explain 59.7% of the variation in the dataset, separate the embryos from eggs injected with the A4+T on both days (hormone‐hormone, red) and the embryos from eggs injected with oil on both days (oil‐oil, purple), but the hormone‐oil (green) and oil‐hormone (blue) injected eggs produced embryos whose size did not sort with LD axes 1, 2, or 3. **Figure S3:** The average heart rate (sampled over 5 minutes) of Japanese Quail (*Coturnix japonica*) embryos on days 9, 12, and 15 of incubation did not differ between embryos in any of the treatment groups (androgen hormones or oil injections on Day 0 and subsequent hormones or oil injections on Day 6; χ^2^_3_=5.16, *P*=0.160). The boxplots depict 10^th^, 25^th^, 50^th^, 75^th^, and 90^th^ percentiles, with all data points shown for each boxplot. **Figure S4:** Twenty‐four hours after the injection of androgen hormones (A4+T) or oil (control) into Japanese Quail (*Coturnix japonica*) eggs on day 0 of incubation. Testosterone concentrations (ng/g) were not statistically elevated in the androgen‐injected eggs relative to the controls (left; *F*_1,8_=0.65, *P*=0.445) and androstenedione concentrations (ng/g) were not elevated in the androgen‐injected eggs relative to the controls (right; *F*_1,8_=1.86, *P*=0.210). The boxplots depict 10^th^, 25^th^, 50^th^, 75^th^, and 90^th^ percentiles, with all data points shown for each boxplot. **Figure S5:** Twenty‐four hours after the injection of androgen hormones (A4+T) or oil (control) into Japanese Quail (*Coturnix japonica*) eggs on day 6 of incubation, testosterone concentrations (ng/g) were not statistically elevated in the androgen‐injected eggs relative to the controls (left; *F*_1,15_=3.35, *P*=0.087) and androstenedione concentrations (ng/g) were not elevated in the androgen‐injected eggs relative to the controls (right; *F*_1,13_=1.15, *P*=0.301). The boxplots depict 10^th^, 25^th^, 50^th^, 75^th^, and 90^th^ percentiles, with all data points shown for each boxplot.

## Data Availability

Data files and the associated analysis code can be found on FigShare. [DOI will not go live until the dataset is published upon manuscript acceptance. Until then, data files can be viewed via a private link, but be aware that author names are attached to the dataset on FigShare: https://figshare.com/s/098480c82879f80822f6].
