# Supplementary material for: Elevating Testosterone and Androstenedione Produces Temporary Sex‐Dependent Variation in Japanese Quail (Coturnix japonica) Embryonic Development
Source: J Exp Zool A Ecol Integr Physiol. 2025 Aug 8;343(9):1103–16. doi: 10.1002/jez.70024 (PMC12484403; doi:10.1002/jez.70024)

# **Supplementary Tables**

**Table S1.** Eigenvalues, percentage of variance explained, and loadings of body parts onto the dimensions of the Day 15 Japanese Quail (*Coturnix japonica*) embryo size principal component analysis.

|  | **PC1** | **PC2** | **PC3** | **PC4** | **PC5** |
| --- | --- | --- | --- | --- | --- |
| Eigenvalues | 3.29 | 0.62 | 0.54 | 0.36 | 0.18 |
| Percentage of Variance | 65.75 | 12.46 | 10.86 | 7.28 | 3.65 |
| **Loadings** |  |  |  |  |  |
| Tarsus length (mm) | 0.70 | 0.20 | 0.02 | 0.00 | 0.08 |
| Bill length (mm) | 0.60 | 0.00 | 0.32 | 0.08 | 0.00 |
| Wing length (mm) | 0.65 | 0.13 | 0.02 | 0.20 | 0.00 |
| Eye diameter (mm) | 0.56 | 0.20 | 0.16 | 0.08 | 0.00 |
| Mass (g) | 0.78 | 0.09 | 0.02 | 0.00 | 0.10 |

**Table S2.** Eigenvalues, percentage of variance, and loadings of egg yolk hormone concentrations (ng/g) onto the dimensions of the Japanese Quail (*Coturnix japonica*) incubation day 15 hormone principal component analysis.

|  | **PC1** | **PC2** | **PC3** | **PC4** | **PC5** |
| --- | --- | --- | --- | --- | --- |
| Eigenvalues | 4.96 | 1.00 | 0.03 | 0.02 | 0.00 |
| Percentage of Variance | 82.65 | 16.60 | 0.47 | 0.29 | 0.00 |
| **Loadings** |  |  |  |  |  |
| Pregnanedione | 0.99 | 0.00 | 0.00 | 0.00 | 0.00 |
| Etiocholanolone | 0.99 | 0.00 | 0.00 | 0.00 | 0.00 |
| Progesterone | 0.99 | 0.00 | 0.00 | 0.00 | 0.00 |
| Pregnenolone | 0.98 | 0.00 | 0.01 | 0.01 | 0.00 |
| 5β-tetrahydrocortisol | 0.01 | 0.99 | 0.00 | 0.00 | 0.00 |
| Pregnanolone | 0.98 | 0.00 | 0.02 | 0.00 | 0.00 |

**Table S3.** Yolk steroid hormones present on sampling days 0 and 1 of incubation. Eggs were injected on Day 0 with either oil or +2 SD A4 and T. Means, standard deviations, and min-max range all ng/gram of yolk.

| **Sampling Day:** | 0 | | 1 | | | |
| --- | --- | --- | --- | --- | --- | --- |
| **Day 0 Injection:** | none | | hormone | | oil | |
|  | **Mean+SD** | **Range** | **Mean + SD** | **Range** | **Mean + SD** | **Range** |
| Androstenedione (A4) | 27.1 + 12.5 | 15.8 - 48.4 | 28.2 + 15.5 | 8.0 - 51.2 | 17.1 + 9.8 | 5.2 - 31.2 |
| Testosterone (T) | 4.9 + 4.2 | 1.9 - 13.3 | 5.0 + 2.7 | 2.1 - 9.0 | 3.6 + 2.8 | 0.7 - 7.7 |
| DHEA | 1.2 + 0.5 | 0.6 - 2.0 | 1.4 + 0.5 | 0.7 - 2.0 | 1.1 + 1.2 | 0.5 - 3.4 |
| Etiocholanolone | 3.8 + 1.8 | 2.1 - 6.3 | 7.0 + 4.3 | 3.0 - 14.2 | 5.3 + 2.3 | 3.0 - 8.2 |
| 11-ketotestosterone | 0.2 + 0.2 | 0.0 - 0.4 | 0.0 + 0.0 | 0.0 - 0.0 | 0.1 + 0.1 | 0.0 - 0.2 |
| Progesterone | 87.6 + 48.7 | 48.2 - 170.6 | 69.9 + 40.5 | 26.6 - 121.8 | 154.2 + 192.1 | 41.2 - 496.0 |
| Pregnenolone | 187.3 + 96.9 | 104.8 - 364.0 | 174.6 + 88.1 | 95.6 - 310.0 | 178.7 + 43.3 | 116.2 - 222.0 |
| 17α-hydroxyprogesterone | 0.0 + 0.1 | 0.0 - 0.2 | 0.0 + 0.0 | 0.0 - 0.0 | 0.2 + 0.5 | 0.0 - 1.1 |
| 17α-hydroxypregnenolone | 10.6 + 1.9 | 7.2 - 12.8 | 11.2 + 2.3 | 8.7 - 13.0 | 10.8 + 2.1 | 8.1 - 14.1 |
| 5β -tetrahydrocortisol | 0.0 + 0.0 | 0.0 - 0.0 | 0.0 + 0.0 | 0.0 - 0.0 | 0.0 + 0.0 | 0.0 - 0.0 |
| Pregnanedione | 1,391.3 + 283.4 | 1,080.0 - 1,726.0 | 1,327.6 + 361.3 | 904.0 - 1,728.0 | 1,361.6 + 205.7 | 1,096.0 - 1,576.0 |
| Pregnanolone | 45.6 + 19.0 | 24.0 - 73.2 | 56.3 + 17.1 | 31.8 - 75.0 | 64.8 + 25.7 | 38.0 - 105.4 |

**Table S4.** Yolk steroid hormones present on sampling day 6 of incubation. Eggs were injected on Day 0 with either oil or +2 SD A4 and T. Means, standard deviations, and min-max range all ng/gram of yolk.

| **Sampling Day** | 6 | | | |
| --- | --- | --- | --- | --- |
| **Day 0 Injection:** | hormone | | oil | |
|  | **Mean + SD** | **Range** | **Mean + SD** | **Range** |
| Androstenedione (A4) | 14.3 + 10.8 | 6.0 - 33.0 | 8.7 + 4.0 | 3.6 - 12.3 |
| Testosterone (T) | 2.4 + 0.7 | 1.6 - 3.2 | 2.3 + 0.9 | 1.4 - 3.5 |
| DHEA | 1.1 + 0.5 | 0.4 - 1.7 | 0.8 + 0.3 | 0.4 - 1.2 |
| Etiocholanolone | 4.1 + 0.8 | 3.6 - 5.4 | 3.6 + 1.4 | 2.2 - 5.2 |
| 11-ketotestosterone | 0.0 + 0.0 | 0.0 - 0.0 | 0.0 + 0.0 | 0.0 - 0.0 |
| Progesterone | 54.4 + 35.6 | 29.4 - 116.6 | 42.6 + 20.0 | 25.6 - 71.6 |
| Pregnenolone | 155.6 + 49.4 | 102.8 - 230.0 | 121.8 + 13.2 | 103.2 - 137.6 |
| 17α-hydroxyprogesterone | 0.0 + 0.0 | 0.0 - 0.0 | 0.0 + 0.0 | 0.0 - 0.0 |
| 17α-hydroxypregnenolone | 7.7 + 2.2 | 5.4 - 10.6 | 6.7 + 2.8 | 3.7 - 11.0 |
| 5β -tetrahydrocortisol | 0.0 + 0.0 | 0.0 - 0.0 | 0.0 + 0.0 | 0.0 - 0.0 |
| Pregnanedione | 1,026.4 + 129.1 | 890.0 - 1,148.0 | 992.4 + 172.0 | 722.0 - 1,174.0 |
| Pregnanolone | 62.4 + 15.1 | 46.2 - 86.2 | 76.0 + 10.1 | 63.6 - 88.4 |

**Table S5.** Yolk steroid hormones present on sampling day 7 of incubation. Eggs were injected on Day 0 and Day 6 with either oil or +2 SD A4 and T. Means, standard deviations, and min-max range all ng/gram of yolk.

| **Sampling Day** | 7 | | | | | | | |
| --- | --- | --- | --- | --- | --- | --- | --- | --- |
| **Injection: D0-D6** | oil-oil | | oil-hormone | | hormone-oil | | hormone-hormone | |
|  | **Mean + SD** | **Range** | **Mean + SD** | **Range** | **Mean + SD** | **Range** | **Mean + SD** | **Range** |
| Androstenedione (A4) | 5.8 + 0.9 | 5.0 - 6.7 | 10.1 + 6.5 | 3.9 - 18.2 | 5.2 + 3.6 | 0.0 - 8.1 | 5.5 + 1.3 | 4.4 - 7.0 |
| Testosterone (T) | 1.2 + 0.5 | 0.6 - 1.6 | 3.6 + 2.4 | 1.4 - 6.2 | 1.6 + 1.0 | 0.4 - 2.4 | 2.0 + 1.2 | 0.6 - 3.7 |
| DHEA | 0.5 + 0.4 | 0.0 - 1.1 | 0.7 + 0.2 | 0.5 - 0.9 | 0.4 + 0.4 | 0.0 - 1.0 | 0.8 + 0.4 | 0.5 - 1.2 |
| Etiocholanolone | 2.5 + 1.0 | 1.5 - 3.9 | 3.7 + 0.9 | 2.6 - 4.6 | 2.8 + 1.1 | 1.5 - 4.0 | 1.9 + 0.6 | 1.4 - 2.8 |
| 11-ketotestosterone | 0.0 + 0.0 | 0.0 - 0.0 | 0.0 + 0.0 | 0.0 - 0.0 | 0.0 + 0.0 | 0.0 - 0.0 | 0.0 + 0.0 | 0.0 - 0.0 |
| Progesterone | 27.8 + 9.4 | 18.4 - 39.8 | 30.3 + 15.4 | 17.1 - 50.2 | 31.5 + 17.8 | 16.0 - 53.8 | 24.5 + 17.3 | 6.5 - 42.8 |
| Pregnenolone | 105.5 + 38.1 | 65.8 - 151.6 | 96.4 + 14.8 | 82.2 - 116.0 | 99.1 + 39.2 | 48.0 - 133.4 | 75.3 + 20.7 | 47.2 - 99.0 |
| 17α-hydroxyprogesterone | 0.0 + 0.0 | 0.0 - 0.0 | 0.0 + 0.0 | 0.0 - 0.0 | 0.0 + 0.0 | 0.0 - 0.0 | 0.0 + 0.0 | 0.0 - 0.0 |
| 17α-hydroxypregnenolone | 4.7 + 1.0 | 3.8 - 5.9 | 6.5 + 1.8 | 4.1 - 8.5 | 5.2 + 0.9 | 4.4 - 6.4 | 2.7 + 2.0 | 0.0 - 5.4 |
| 5β -tetrahydrocortisol | 0.0 + 0.0 | 0.0 - 0.0 | 0.0 + 0.0 | 0.0 - 0.0 | 0.0 + 0.0 | 0.0 - 0.0 | 0.1 + 0.3 | 0.0 - 0.6 |
| Pregnanedione | 632.5 + 104.6 | 502.0 - 758.0 | 762.0 + 103.4 | 650.0 - 892.0 | 774.0 + 271.9 | 506.0 - 1,024.0 | 488.0 + 139.8 | 312.0 - 654.0 |
| Pregnanolone | 57.5 + 12.7 | 41.2 - 70.2 | 52.2 + 3.6 | 48.2 - 55.2 | 64.1 + 30.1 | 37.2 - 103.6 | 60.9 + 26.3 | 25.8 - 88.2 |

**Table S6.** Yolk steroid hormones present on sampling day 15 of incubation. Eggs were injected on Day 0 and Day 6 with either oil or +2 SD A4 and T. Means, standard deviations, and min-max range all ng/gram of yolk.

| **Sampling Day** | 15 | | | | | | | |
| --- | --- | --- | --- | --- | --- | --- | --- | --- |
| **Injection: D0-D6** | oil-oil | | oil-hormone | | hormone-oil | | hormone-hormone | |
|  | **Mean + SD** | **Range** | **Mean + SD** | **Range** | **Mean + SD** | **Range** | **Mean + SD** | **Range** |
| Androstenedione (A4) | 0.0 + 0.0 | 0.0 - 0.0 | 0.0 + 0.0 | 0.0 - 0.0 | 0.0 + 0.0 | 0.0 - 0.0 | 0.0 + 0.0 | 0.0 - 0.0 |
| Testosterone (T) | 0.0 + 0.0 | 0.0 - 0.0 | 0.0 + 0.0 | 0.0 - 0.0 | 0.0 + 0.0 | 0.0 - 0.0 | 0.0 + 0.0 | 0.0 - 0.0 |
| DHEA | 0.0 + 0.0 | 0.0 - 0.0 | 0.0 + 0.0 | 0.0 - 0.0 | 0.0 + 0.0 | 0.0 - 0.0 | 0.0 + 0.0 | 0.0 - 0.0 |
| Etiocholanolone | 0.0 + 0.0 | 0.0 - 0.0 | 0.0 + 0.0 | 0.0 - 0.0 | 0.2 + 0.4 | 0.0 - 0.8 | 0.0 + 0.0 | 0.0 - 0.0 |
| 11-ketotestosterone | 0.0 + 0.0 | 0.0 - 0.0 | 0.0 + 0.0 | 0.0 - 0.0 | 0.0 + 0.0 | 0.0 - 0.0 | 0.0 + 0.0 | 0.0 - 0.0 |
| Progesterone | 0.0 + 0.0 | 0.0 - 0.1 | 0.0 + 0.1 | 0.0 - 0.1 | 5.5 + 11.8 | 0.0 - 26.6 | 0.1 + 0.1 | 0.0 - 0.2 |
| Pregnenolone | 3.9 + 2.1 | 1.1 - 7.0 | 3.7 + 1.8 | 2.4 - 6.6 | 11.2 + 17.7 | 1.6 - 42.8 | 2.6 + 0.8 | 1.3 - 3.4 |
| 17α-hydroxyprogesterone | 0.0 + 0.0 | 0.0 - 0.0 | 0.0 + 0.0 | 0.0 - 0.0 | 0.0 + 0.0 | 0.0 - 0.0 | 0.0 + 0.0 | 0.0 - 0.0 |
| 17α-hydroxypregnenolone | 0.0 + 0.0 | 0.0 - 0.0 | 0.0 + 0.0 | 0.0 - 0.0 | 0.0 + 0.0 | 0.0 - 0.0 | 0.0 + 0.0 | 0.0 - 0.0 |
| 5β -tetrahydrocortisol | 0.6 + 1.3 | 0.0 - 3.0 | 0.1 + 0.3 | 0.0 - 0.6 | 0.1 + 0.1 | 0.0 - 0.3 | 0.2 + 0.3 | 0.0 - 0.5 |
| Pregnanedione | 1.9 + 0.6 | 1.2 - 2.6 | 1.8 + 0.5 | 1.0 - 2.3 | 77.2 + 158.2 | 2.1 - 360.0 | 2.4 + 0.3 | 2.1 - 2.7 |
| Pregnanolone | 0.3 + 0.1 | 0.1 - 0.3 | 0.4 + 0.1 | 0.2 - 0.6 | 7.6 + 12.8 | 0.4 - 30.0 | 0.5 + 0.2 | 0.2 - 0.8 |

| **Response Variable** | **Predictors** | **η^2^** | ***F_3,12_*** | ***P*** |
| --- | --- | --- | --- | --- |
| Embryo PC1 | Hormone PC1 | 0.10 | 2.40 | 0.147 |
|  | Injection | 0.20 | 1.62 | 0.236 |
|  | Interaction | 0.20 | 1.63 | 0.234 |
| Embryo PC1 | Hormone PC2 | 0.08 | 1.54 | 0.238 |
|  | Injection | 0.23 | 1.42 | 0.283 |
|  | Interaction | 0.07 | 0.47 | 0.711 |
| Embryo PC2 | Hormone PC1 | 0.00 | 0.01 | 0.942 |
|  | Injection | 0.40 | 3.29 | 0.058 |
|  | Interaction | 0.10 | 0.79 | 0.521 |
| Embryo PC2 | Hormone PC2 | 0.00 | 0.05 | 0.832 |
|  | Injection | 0.41 | 2.87 | 0.081 |
|  | Interaction | 0.01 | 0.09 | 0.963 |
|  | | **η^2^** | ***F_1,16_*** | ***P*** |
| Embryo PC1 | Hormone PC1 | 0.12 | 2.50 | 0.134 |
|  | Sex | 0.08 | 1.61 | 0.223 |
|  | Interaction | 0.03 | 0.61 | 0.446 |
| Embryo PC1 | Hormone PC2 | 0.03 | 0.55 | 0.468 |
|  | Sex | 0.03 | 0.56 | 0.465 |
|  | Interaction | 0.00 | 0.001 | 0.964 |
| Embryo PC2 | Hormone PC1 | 0.00 | 0.05 | 0.828 |
|  | Sex | 0.08 | 1.44 | 0.247 |
|  | Interaction | 0.04 | 0.69 | 0.417 |
| Embryo PC2 | Hormone PC2 | 0.00 | 0.004 | 0.951 |
|  | Sex | 0.08 | 1.44 | 0.248 |
|  | Interaction | 0.01 | 0.19 | 0.666 |
|  | | **η^2^** | ***F_1,18_*** | ***P*** |
| Hormone PC1 | Sex | 0.02 | 0.38 | 0.544 |
| Hormone PC2 | Sex | 0.07 | 1.35 | 0.261 |
|  | | **η^2^** | ***F_1,12_*** | ***P*** |
| Hormone PC1 | Sex | 0.04 | 0.79 | 0.391 |
|  | Injection | 0.22 | 1.52 | 0.262 |
|  | Interaction | 0.17 | 1.16 | 0.367 |
| Hormone PC2 | Sex | 0.09 | 1.78 | 0.207 |
|  | Injection | 0.12 | 0.81 | 0.512 |
|  | Interaction | 0.23 | 1.62 | 0.237 |

**Table S7.** Statistical models of Japanese Quail (*Coturnix japonica*) embryo body size principal components, egg yolk steroid hormone principal components, injection treatment (Day 0 and Day 6 hormone or oil injections), and embryo chromosomal sex on Day 15 of incubation.

# **Supplementary Figures**

**Figure S1.** Linear discriminant (LD) scores illustrate an interaction between the effect of embryonic sex and androgen hormone injection on the size of 6-day old Japanese Quail (*Coturnix japonica*) embryos. LD1 (x-axis) scores define a sex × treatment interaction, while LD2 (y-axis) scores show a sex effect on body size.
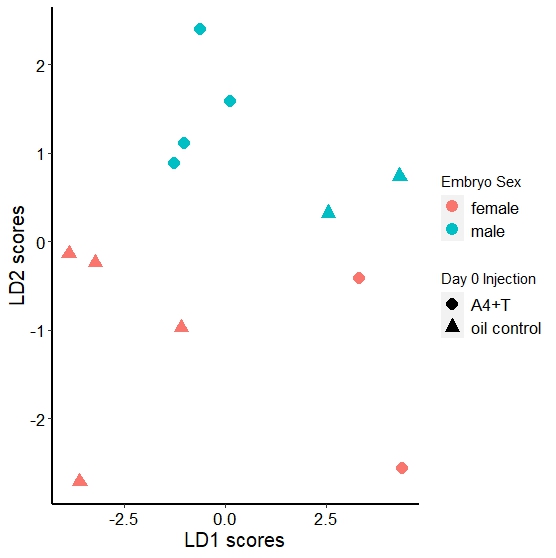


**Figure S2.** Linear discriminant (LD) scores for LD 1 (x-axis, both plots), 2 (y-axis, left), and 3 (y-axis, right) show little separation in Japanese Quail (*Coturnix japonica*) embryonic body size measurements on Day 15 of incubation following androgen hormone or oil control egg injections (on both Days 0 and 6 of incubation). LD1 scores, which explain 59.7% of the variation in the dataset, separate the embryos from eggs injected with the A4+T on both days (hormone-hormone, red) and the embryos from eggs injected with oil on both days (oil-oil, purple), but the hormone-oil (green) and oil-hormone (blue) injected eggs produced embryos whose size did not sort with LD axes 1, 2, or 3.
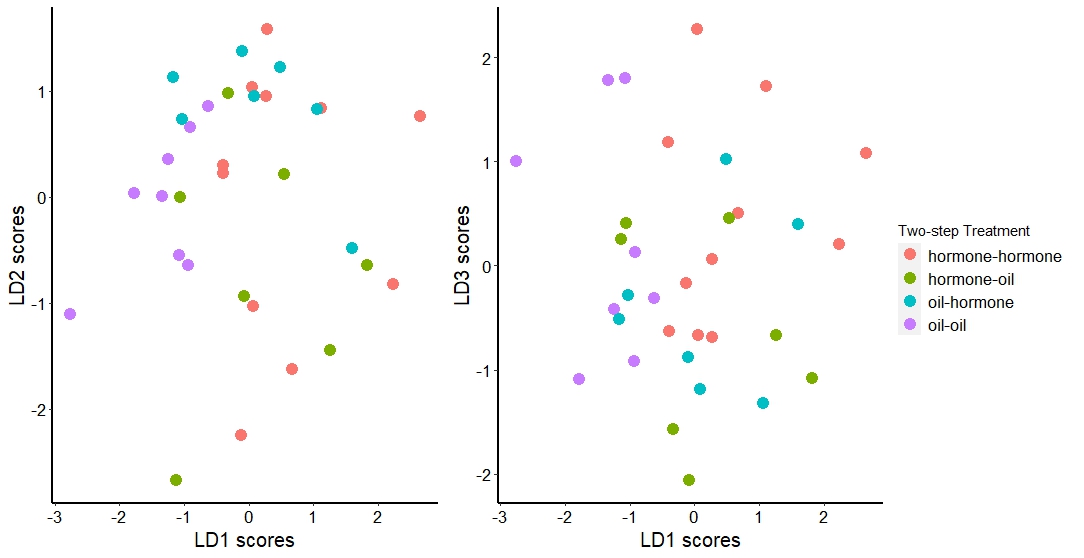


**Figure S3.** The average heart rate (sampled over 5 minutes) of Japanese Quail (*Coturnix japonica*) embryos on days 9, 12, and 15 of incubation did not differ between embryos in any of the treatment groups (androgen hormones or oil injections on Day 0 and subsequent hormones or oil injections on Day 6; χ^2^_3_=5.16, *P*=0.160). The boxplots depict 10^th^, 25^th^, 50^th^, 75^th^, and 90^th^ percentiles, with all data points shown for each boxplot.


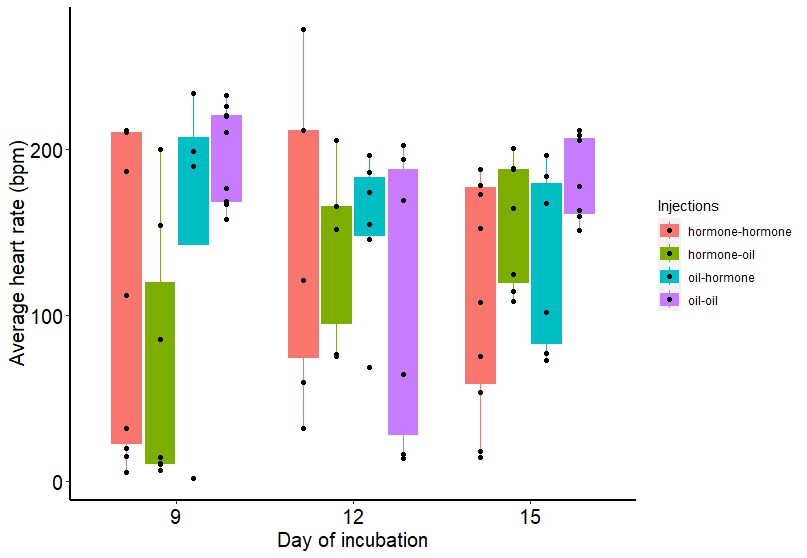


**Figure S4.** Twenty-four hours after the injection of androgen hormones (A4+T) or oil (control) into Japanese Quail (*Coturnix japonica*) eggs on day 0 of incubation. Testosterone concentrations (ng/g) were not statistically elevated in the androgen-injected eggs relative to the controls (left; *F*_1,8_=0.65, *P*=0.445) and androstenedione concentrations (ng/g) were not elevated in the androgen-injected eggs relative to the controls (right; *F*_1,8_=1.86, *P*=0.210). The boxplots depict 10^th^, 25^th^, 50^th^, 75^th^, and 90^th^ percentiles, with all data points shown for each boxplot.


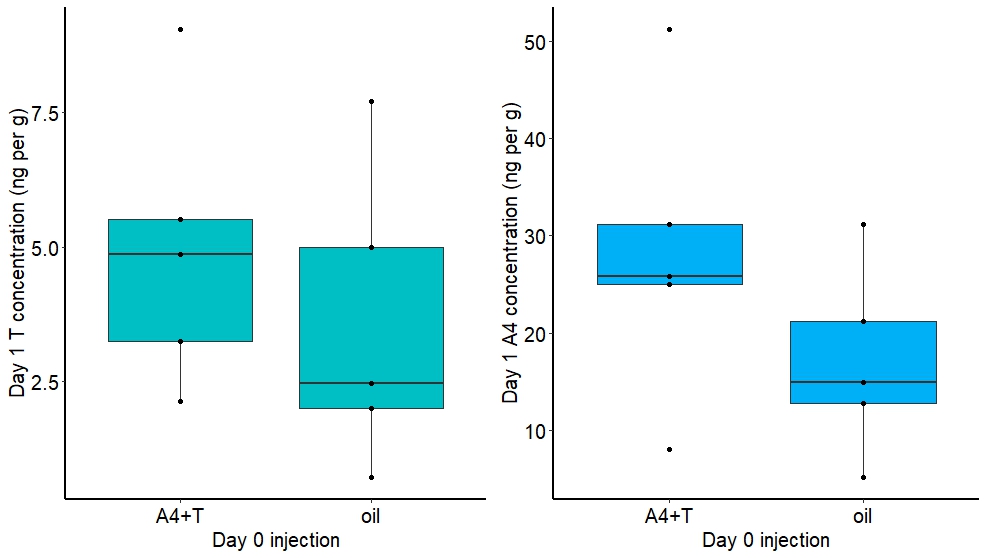


**Figure S5.** Twenty-four hours after the injection of androgen hormones (A4+T) or oil (control) into Japanese Quail (*Coturnix japonica*) eggs on day 6 of incubation, testosterone concentrations (ng/g) were not statistically elevated in the androgen-injected eggs relative to the controls (left; *F*_1,15_=3.35, *P*=0.087) and androstenedione concentrations (ng/g) were not elevated in the androgen-injected eggs relative to the controls (right; *F* _1,13_=1.15, *P*=0.301). The boxplots depict 10^th^, 25^th^, 50^th^, 75^th^, and 90^th^ percentiles, with all data points shown for each boxplot.


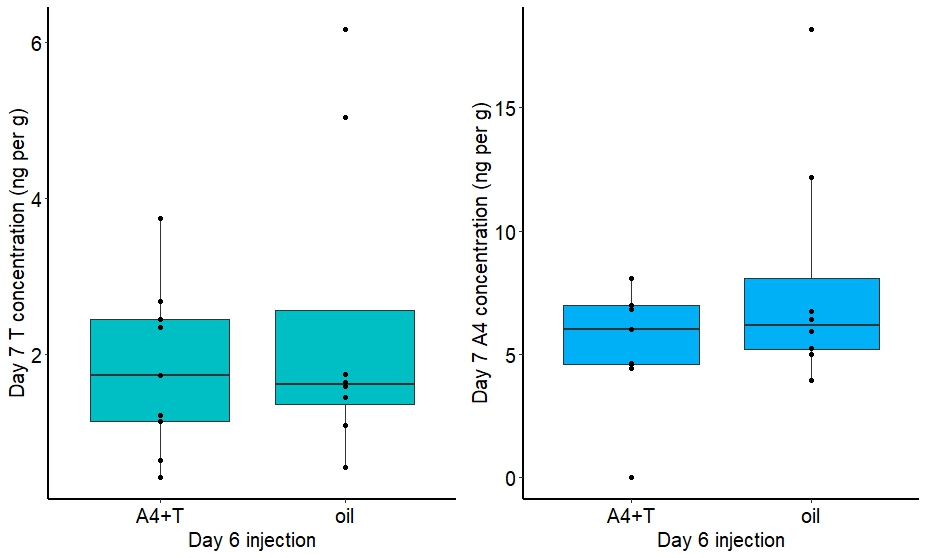

Supplement: Supplementary file 1 — Table S1: Eigenvalues, percentage of variance explained, and loadings of body parts onto the dimensions of the Day 15 Japanese Quail (Coturnix japonica) embryo size principal component analysis. Table S2: Eigenvalues, percentage of variance, and loadings of egg yolk hormone concentrations (ng/g) onto the dimensions of the Japanese Quail (Coturnix japonica) incubation day 15 hormone principal component analysis. Table S3: Yolk steroid hormones present on sampling days 0 and 1 of incubation. Eggs were injected on Day 0 with either oil or +2 SD A4 and T. Means, standard deviations, and min‐max range all ng/gram of yolk. Table S4: Yolk steroid hormones present on sampling day 6 of incubation. Eggs were injected on Day 0 with either oil or +2 SD A4 and T. Means, standard deviations, and min‐max range all ng/gram of yolk. Table S5: Yolk steroid hormones present on sampling day 7 of incubation. Eggs were injected on Day 0 and Day 6 with either oil or +2 SD A4 and T. Means, standard deviations, and min‐max range all ng/gram of yolk. Table S6: Yolk steroid hormones present on sampling day 15 of incubation. Eggs were injected on Day 0 and Day 6 with either oil or +2 SD A4 and T. Means, standard deviations, and min‐max range all ng/gram of yolk. Table S7: Statistical models of Japanese Quail (Coturnix japonica) embryo body size principal components, egg yolk steroid hormone principal components, injection treatment (Day 0 and Day 6 hormone or oil injections), and embryo chromosomal sex on Day 15 of incubation. Figure S1: Linear discriminant (LD) scores illustrate an interaction between the effect of embryonic sex and androgen hormone injection on the size of 6‐day old Japanese Quail (Coturnix japonica) embryos. LD1 (x‐axis) scores define a sex × treatment interaction, while LD2 (y‐axis) scores show a sex effect on body size. Figure S2: Linear discriminant (LD) scores for LD 1 (x‐axis, both plots), 2 (y‐axis, left), and 3 (y‐axis, right) show little separation in Japanese Quail [file JEZ-343-1103-s001.docx]
